# Comparison of the effect of quercetin and daidzein on production performance, anti-oxidation, hormones, and cecal microflora in laying hens during the late laying period

**DOI:** 10.1016/j.psj.2023.102674

**Published:** 2023-03-27

**Authors:** Jiayan Liu, Yuxin Fu, Shuaishuai Zhou, Pengyu Zhao, Jian Zhao, Qinglin Yang, Hao Wu, Manyi Ding, Yao Li

**Affiliations:** ⁎ College of Animal Science and Technology, Northeast Agricultural University, Harbin, 150030, China; † Hebei Innovative Technology Center of Plant-derived Animal Nutrients, Handan, 057250, China; ‡ Chenguang Biotech Group Co., Ltd, Handan, 057250, China

**Keywords:** quercetin, daidzein, late laying period, laying hen, production performance

## Abstract

This study aims to compare the effect of quercetin and daidzein on production performance, anti-oxidation, hormones, and cecal microflora in laying hens during the late laying period. A total of 360 53-week-old healthy Hyline brown laying hens were randomly divided into 3 groups (control, 0.05% quercetin, and 0.003% daidzein). Diets were fed for 10 wk, afterwards 1 bird per replicate (6 replicates) were euthanized for sampling blood, liver and cecal digesta. Compared with the control, quercetin significantly increased laying rate and decreased feed-to-egg weight ratio from wk 1 to 4, wk 5 to 10, and wk 1 to 10 (*P* < 0.05). Quercetin significantly increased the activities of superoxide dismutase (**SOD**) and glutathione peroxidase (**GSH-Px**) and decreased catalase (**CAT**) activity and malondialdehyde (**MDA**) content in serum and liver (*P* < 0.05) and increased content of total antioxidant capacity (**T-AOC**) in liver (*P* < 0.05). Quercetin increased content of estradiol (**E_2_**), luteinizing hormone (**LH**), follicle-stimulating hormone (**FSH**), growth hormone (**GH**), insulin-like growth factor 1 (**IGF-1**), triiodothyronine (**T_3_**) and thyroxine (**T_4_**) in serum (*P* < 0.05). Quercetin significantly decreased the relative abundance of *Bacteroidaceae* and *Bacteroides* (*P* < 0.01) and significantly increased the relative abundance of *Lactobacillaceae* and *Lactobacillus* (*P* < 0.05) at family and genus levels in cecum. Daidzein did not significantly influence production performance from wk 1 to 10. Daidzein significantly increased SOD activity and decreased CAT activity and MDA content in serum and liver (*P* < 0.05), and increased T-AOC content in liver (*P* < 0.05). Daidzein increased content of FSH, IGF-1, T_3_ in serum (*P* < 0.05). Daidzein increased the relative abundance of *Rikenellaceae RC9 gut group* at genus level in cecum (*P* < 0.05). Quercetin increased economic efficiency by 137.59% and 8.77%, respectively, compared with daidzein and control. In conclusion, quercetin improved production performance through enhancing antioxidant state, hormone levels, and regulating cecal microflora in laying hens during the late laying period. Quercetin was more effective than daidzein in improving economic efficiency.

## INTRODUCTION

In recent years, laying hens farming has been expanded to meet the needs of human life in China. Egg production ranks first and the per capita consumer of eggs is about 1.7 times the world level (Yang et al., 2019). The egg production cycle of commercial laying hens are divided into 3 stages: early laying period, peak laying period, and late laying period. The late laying period of laying hens refers to when the laying rate is less than 80% after 48-week-old and accounts for about half of the production cycle (Wang et al., 2017). Moreover, oxidative damage, abnormal hormone secretion, and immunosuppression decrease production performance in laying hens during the late laying period. Therefore, how to improve production performance using nutritional regulation is of importantly significant in laying hens during the late laying period. Recently, safe additive has been used for improving animal production due to banning antibiotics in feed, including vitamins (Gan et al., 2020), minerals (Dalia et al., 2018), probiotics (Lv et al., 2022), and flavonoids (Zhao et al., 2017). Now supplementation with dietary flavonoids is an important way to improve production performance in laying hens during the late laying period.

Quercetin is a polyhydroxy flavonoid, belonging to flavonols, which is rich in onion, hawthorn, sea buckthorn, apple, *Ginkgo biloba*, and other plants. It has a variety of biological activities, including anti-oxidation, anti-bacteria, anti-virus, etc. (Azeem et al., 2022; Nguyen and Bhattacharya, 2022), which is low price, high safety, extensive sources, and ubiquitously applied in the field of medicine and animal husbandry. Previous studies of our research team have shown that diet supplemented with 0.04% quercetin improved production performance in laying hens (Jin, 2013; Hu, 2014; Sun, 2015). Daidzein is one of the isoflavones, which exists mostly in legumes (Krizova et al., 2019). It is commonly used in the medical field, potentially treating human diseases, such as breast cancer, cardiovascular diseases, and menopause (Atkinson et al., 2005). Recent studies have shown that daidzein may promote growth and production performance in birds because of anti-oxidation, a weak estrogen, and anti-estrogen, immune regulation, other physiological and biochemical activities (Ni et al., 2007; Lu et al., 2017).

Both quercetin and daidzein may improve production performance in laying hens, however, the high price of daidzein limits its uses in animal production. If the effect of quercetin on production performance is similar to daidzein in laying hens during the late laying period, quercetin substitutes for daidzein will decrease the feeding cost and increase economic efficiency. Therefore, this study compared the effect of diet supplemented with separate quercetin and daidzein on production performance, anti-oxidation, hormones, and cecal microflora in laying hens during the late laying period, this will provide a scientific basis for applying quercetin in hen production.

## MATERIALS AND METHODS

### Experimental Animals and Diets

A total of 360 healthy 53-week-old Hyline Brown laying hens (purchased from Harbin Yinong Poultry Industry, Harbin, China) with similar body weight (2.00 ± 0.05 kg) and laying rate (80.44% ± 0.79%) were randomly assigned to 3 groups (120 birds each group) consisting of 6 replicates of 20 birds each replicate. The control group was fed a corn-soybean basal diet according to Chinese Layers Feeding Standards (Table 1, GB/T 5916-2020), 2 experimental groups were fed the corn-soybean basal diet supplemented with 0.05% quercetin and 0.003% daidzein, respectively. Quercetin (purity ≥ 97%) and daidzein (purity ≥ 98%) used in this study was purchased from Nanjing Dulai Biotechnology Co. Ltd. (Nanjing, China) and Meryer (Shanghai) Chemical Technology Co. Ltd. (Shanghai, China), respectively. The laying hens were housed in fully folded 3-dimensional wire cages under natural light and artificial light (7 W LED lamp, 16 h light per day), and maintained optimal ventilation during the experimental period. The adaptation period was 1 wk. The trial lasted for 10 wk.

**Table 1:** Composition and nutrient composition of basal diet (air-dried basal diet).

Ingredient	Content (%)	Nutrient levels	Calculated value
Corn meal	60.00	ME(MJ/kg)	10.72
Soybean meal	22.00	CP(%)	15.22
Calcium bicarbonate	1.58	CEE(%)	2.81
Wheat bran	5.00	CF(%)	2.54
Limestone	7.90	Ca(%)	3.33
Sodium chloride	0.32	TP(%)	0.35
Methionine	0.20	Lys(%)	0.75
Vitamin and Mineral premix	3.00	Met+Cys(%)	0.49
Total	100.00		

Abbreviations: CEE, crude ether extract; CF, crude fiber; CP, crude protein; Cys, cysteine; Lys, lysine; ME, metabolic energy; Met, methionine; TP, total phosphorus.

Per-kilogram of diet provides:10,000 IU of vitamin A, 33,000 IU of vitamin D_3_, 21 IU of vitamin E, 3 mg of vitamin K_3_, 2 mg of vitamin B_1_, 8 mg of vitamin B_2_, 4 mg of vitamin B_6_, 0.02 mg of vitamin B_12_, 37.2 mg of nicotinic acid, 10.8 mg of pantothenic acid, 1 mg of folic acid, 0.1mg of d-biotin, 0.64 g of choline chloride, 8 mg of I (KI), 0.5 mg of Se (NaSeO_3_), 65 mg of Fe (FeSO_4_·7H_2_O), 120 mg of Mn (MnSO_4_·H_2_O), 10 mg of Cu (MnSO_4_·H_2_O), 65 mg of Zn (ZnO). Values of nutrient levels were calculated.

### Production Performance

The number of eggs, egg weight, feed intake, and remaining feed were recorded each day. The average laying rate, egg weight, daily feed intake, and feed-to-egg weight ratio of each group were calculated. Laying rate (%) = egg production / number of birds × 100%. Feed-to-egg weight ratio = daily feed consumption / average egg weight.

### Sample Preparation

At the end of the 10th wk of the experiment, 1 bird per replicate (Guo et al., 2020b) (6 replicates) were randomly selected and euthanized by cervical dislocation. The jugular vein blood samples were collected in 10 mL centrifuge tubes, standing for 2 to 3 h, and centrifuging at 3000 r/min for 15 min. Serum was collected and stored at -80 ℃. After blood collection, the chickens were dissected, 5 to 8 cm liver and fresh cecal digesta were taken, quickly frozen in liquid nitrogen, and stored at -80 ℃. Before the determination, the liver was prepared with an appropriate saline by homogenate. The supernatant was obtained by centrifuging at 3000 r/min for 10 min.

### Indices of Anti-Oxidation and Hormones

The activities of catalase (**CAT**), superoxide dismutase (**SOD**), glutathione peroxidase (**GSH-Px**) and content of total antioxidant capacity (**T-AOC**), malondialdehyde (**MDA**) in serum and liver, content of estradiol (**E_2_**), luteinizing hormone (**LH**), follicle-stimulating hormone (**FSH**), growth hormone (**GH**), insulin-like growth factor 1 (**IGF-1**), triiodothyronine (**T_3_**), thyroxine (**T_4_**) in serum were determined using enzyme-labeled instrument according to enzyme-linked immunosorbent assay (**ELISA**) kit instructions purchased from Jiangsu Baolai Biotechnology Co. Ltd. (Jiangsu, China).

### Cecal Microflora

The SDS method was used to extract DNA from cecal digesta. Primers 515F 5*'*-GTGCCAGCMGCCGCGGTAA-3*'* and 806R 5*'*-GGACTACHVGGGTWTCTAAT-3′ containing specific bands were used to amplify the V4 region of 16S rDNA (Guo et al., 2022). The library was constructed and sequenced by Wuh-an GeneCreate Biological Engineering Co. Ltd. (Wuhan, China) using the Illu-mina NovaSeq platform.

### Statistical Analysis

The data from this experiment were analyzed by One-way ANOVA of SPSS 26.0 software and Duncan's method was used for multiple comparisons. All the results were expressed as the “Means ± SEM,” *P* < 0.05 means significant difference, and *P* > 0.05 represents an insignificant difference. The GraphPad Prism 9.5 software was used to draw the histogram.

Analysis of microbiome: the Raw Data obtained from sequencing were spliced and filtered to obtain Clear Data. Operational taxonomic units (**OTUs**) clustering and species classification analysis were conducted based on Clear Data. According to taxonomic information, species richness, common and endemic OTUs and community structure differences in different samples and groups were obtained. The economic efficiency only included feeding costs under the same other conditions in the experiment. The basal diet was 2800.00 CNY/t, the quercetin was 1.00 CNY/g, the daidzein was 22.24 CNY/g, and the price of eggs was 9.8 CNY/kg at the end of the trial.

## RESULTS

Compared with the control, quercetin significantly increased laying rate (*P* < 0.05) and decreased feed-to-egg weight ratio (*P* < 0.05) from wk 1 to 4, wk 5 to 10, and wk 1 to 10; daidzein did not significantly affect laying rate and feed-to-egg weight ratio from wk 1 to 10. There was no significant difference among 3 groups in average egg weight and average daily feed intake from wk 1 to 10 . Compared with the daidzein, quercetin significantly increased laying rate (*P* < 0.05) and decreased feed-to-egg weight ratio (*P* < 0.05) from wk 1 to 4, wk 1 to 10. Compared with the control and daidzein, quercetin increased laying rate by 11.97% and 8.91%, decreased feed-to-egg weight ratio by 10.96% and 7.80% from wk 1 to 10, respectively (Table 2).

**Table 2:** Effects of quercetin and daidzein on production performance in laying hens during the late laying period.

Items	Control	Quercetin	Daidzein	SEM	*P* value
Laying rate (%)					
Wk 1–4	74.98^b^	83.73^a^	74.75^b^	0.83	<0.01
Wk 5–10	67.41^b^	75.65^a^	71.16^ab^	3.23	0.074
Wk 1–10	70.77^b^	79.24^a^	72.76^b^	2.41	0.005
Average egg weight (g)					
Wk 1–4	64.66	63.93	62.88	0.81	0.141
Wk 5–10	62.91	63.57	63.26	0.75	0.685
Wk 1–10	63.69	63.73	63.09	0.58	0.475
Average daily feed intake (g)					
Wk 1–4	132.07	131.67	131.71	3.43	0.992
Wk 5–10	126.93	127.28	127.06	2.03	0.985
Wk 1–10	129.22	129.23	129.12	2.12	0.999
Feed-to-egg weight ratio^1^					
Wk 1–4	2.71^a^	2.49^b^	2.77^a^	0.06	0.003
Wk 5–10	3.09^a^	2.69^b^	2.86^ab^	0.12	0.017
Wk 1–10	2.92^a^	2.60^b^	2.82^a^	0.09	0.006

a,b Means in the same row with different letters are significantly different (*P* < 0.05). n = 6.

1 Feed-to-egg weight ratio was calculated as: feed-to-egg weight ratio = daily feed consumption/average egg weight.

Compared with the control, quercetin and daidzein significantly decreased MDA content (*P* < 0.05) and CAT activity (*P* < 0.05), and increased SOD activity (*P* < 0.05) in serum; quercetin significantly increased GSH-Px activity (*P* < 0.05). Compared with the daidzein, quercetin significantly increased activities of GSH-Px and CAT (*P* < 0.05) and decreased SOD activity (*P* < 0.05) in serum (Table 3).

**Table 3:** Effects of quercetin and daidzein on antioxidant indices in serum of laying hens during the late laying period.

Items	Control	Quercetin	Daidzein	SEM	*P* value
MDA (nmol/mL)	11.17^a^	8.34^b^	7.87^b^	0.53	<0.01
SOD (U/mL)	225.37^c^	330.51^b^	368.96^a^	17.60	<0.01
GSH-Px (U/mL)	141.68^b^	183.59^a^	154.47^b^	6.95	<0.01
CAT (U/mL)	85.08^a^	67.54^b^	57.70^c^	3.73	<0.01
T-AOC (ng/mL)	12.29	13.25	12.92	0.81	0.504

Abbreviations: CAT, catalase; GSH-Px, glutathione peroxidase; MDA, malondialdehyde; SOD, superoxide dismutase; T-AOC, total antioxidant capacity.

a,b,c Means in the same row with different letters are significantly different (*P* < 0.05). n = 6.

Compared with the control, quercetin and daidzein significantly decreased MDA content (*P* < 0.05) and CAT activity (*P* < 0.05), significantly increased SOD activity (*P* < 0.05) and T-AOC content (*P* < 0.05) in liver; quercetin significantly enhanced GSH-Px activity (*P* < 0.05). Compared with the daidzein, quercetin significantly increased MDA content (*P* < 0.05) and GSH-Px activity (*P* < 0.05) in liver (Table 4).

**Table 4:** Effects of quercetin and daidzein on antioxidant indices in liver of laying hens during the late laying period.

Items	Control	Quercetin	Daidzein	SEM	*P* value
MDA (nmol/g)	137.09^a^	106.23^b^	86.62^c^	4.07	<0.01
SOD (U/g)	1883.08^b^	3177.84^a^	3289.17^a^	234.15	<0.01
GSH-Px (U/g)	1236.15^b^	1662.64^a^	1301.28^b^	75.18	<0.01
CAT (U/g)	691.14^a^	504.90^b^	481.83^b^	29.35	<0.01
T-AOC (ng/g)	89.20^b^	110.37^a^	117.43^a^	7.65	0.006

Abbreviations: CAT, catalase; GSH-Px, glutathione peroxidase; MDA, malondialdehyde; SOD, superoxide dismutase; T-AOC, total antioxidant capacity.

a,b,c Means in the same row with different letters are significantly different (*P* < 0.05). n = 6.

Compared with the control, quercetin significantly increased content of E_2_ (*P* < 0.05), LH (*P* < 0.05) and FSH (*P* < 0.05) in serum; daidzein significantly increased FSH content (*P* < 0.05). Compared with the daidzein, quercetin significantly enhanced content of E_2_ (*P* < 0.05) and LH (*P* < 0.05) in serum (Table 5).

**Table 5:** Effects of quercetin and daidzein on content of reproductive hormones in serum of laying hens during the late laying period.

Items	Control	Quercetin	Daidzein	SEM	*P* value
E_2_ (pmol/L)	34.26^b^	57.10^a^	36.75^b^	2.51	<0.01
LH (ng/mL)	5.03^b^	12.05^a^	5.82^b^	0.49	<0.01
FSH (mIU/mL)	12.38^b^	22.63^a^	22.71^a^	1.21	<0.01

Abbreviations: E_2_, estradiol; FSH, follicle-stimulating hormone; LH, luteinizing hormone.

a,b Means in the same row with different letters are significantly different (*P* < 0.05). n = 6.

Compared with the control, quercetin significantly increased content of GH (*P* < 0.05), IGF-1 (*P* < 0.05), T_3_ (*P* < 0.05), and T_4_ (*P* < 0.05) in serum; daidzein significantly increased content of IGF-1 (*P* < 0.05) and T_3_ (*P* < 0.05) in serum. Compared with the daidzein, quercetin significantly increased content of GH (*P* < 0.05), IGF-1 (*P* < 0.05), T_3_ (*P* < 0.05), and T_4_ (*P* < 0.05) in serum (Table 6).

**Table 6:** Effects of quercetin and daidzein on content of growth hormones in serum of laying hens during the late laying period.

Items	Control	Quercetin	Daidzein	SEM	*P* value
GH (ng/mL)	20.71^b^	24.35^a^	22.15^b^	0.79	0.001
IGF-1 (ng/mL)	146.91^c^	197.66^a^	176.11^b^	8.69	<0.01
T_4_ (ng/mL)	146.44^b^	219.68^a^	164.22^b^	11.92	<0.01
T_3_ (ng/mL)	5.92^c^	8.69^a^	6.75^b^	0.37	<0.01

Abbreviations: GH, growth hormone; IGF-1, insulin-like growth factor 1; T_4_, thyroxine; T_3_, triiodothyronine.

a,b,c Means in the same row with different letters are significantly different (*P* < 0.05). n = 6.

**Figure 1:**
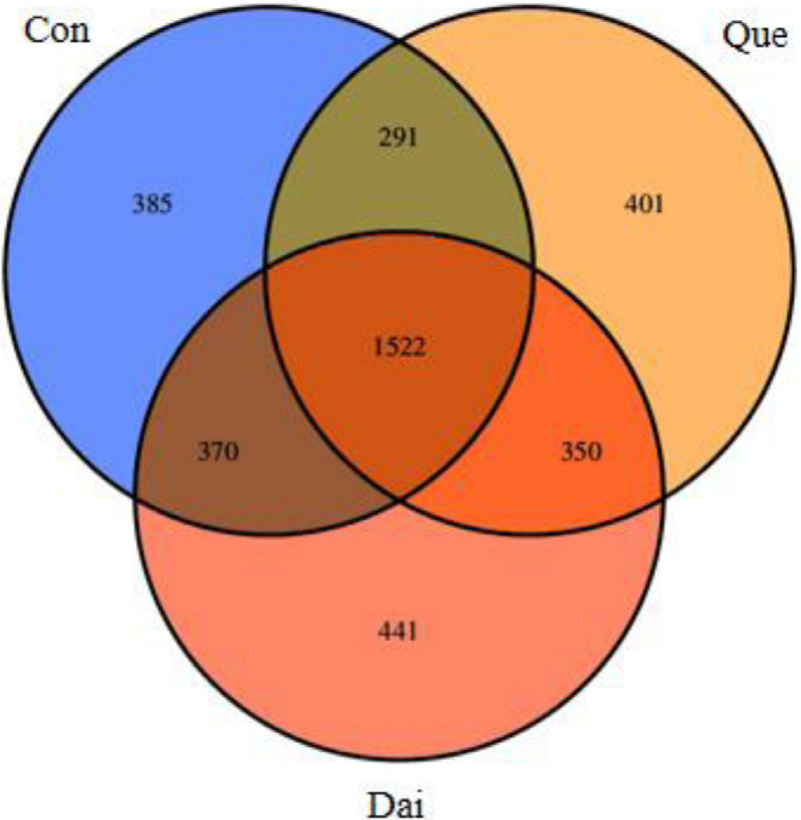
Venn diagram of OTU in cecal microflora of laying hens during the late laying period. Con, Que and Dai represented control, 0.05% quercetin group and 0.003% daidzein group, respectively. n = 6. Abbreviation: OTU, operational taxonomic units.

There were 1522 OTUs in the 3 groups of microflora in cecum, accounting for 40.48%. The specific OTUs of the control group, quercetin group and daidzein group were 385, 401, and 441, respectively (Figure 1).

**Figure 2:**
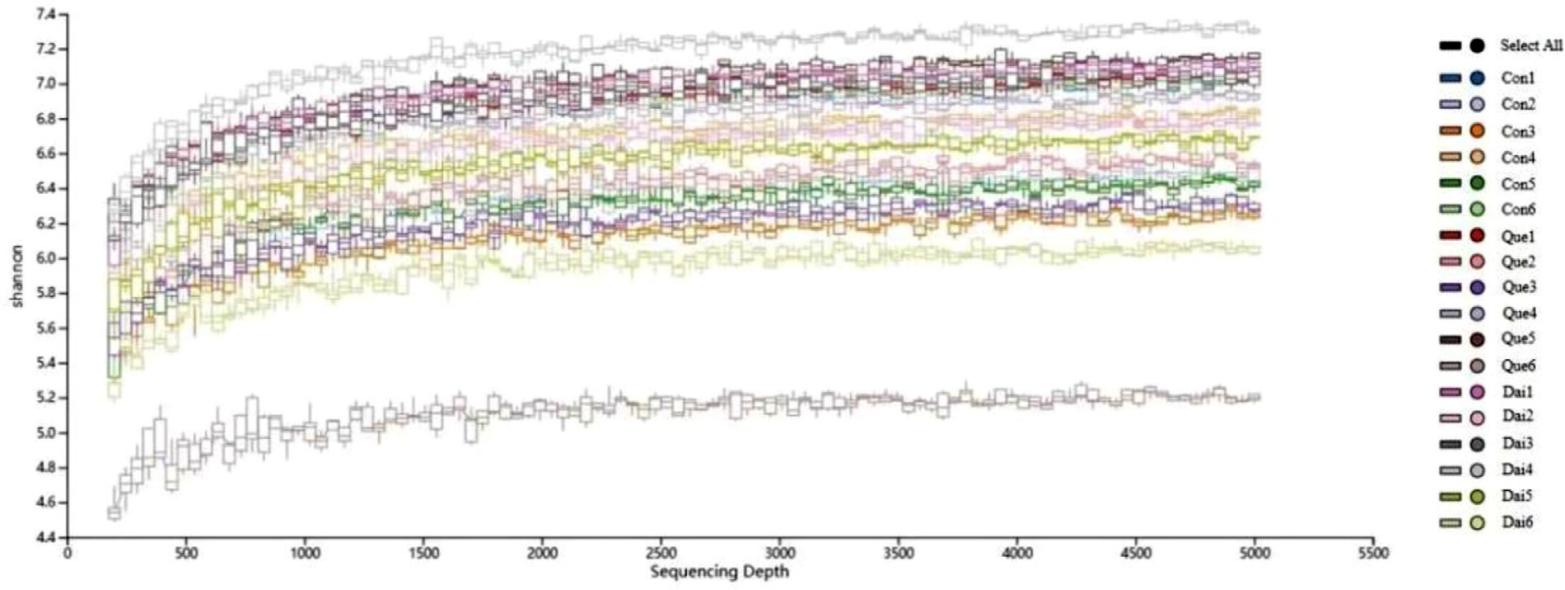
Shannon-Wiener curves of cecal microflora in laying hens during the late laying period. Con, Que and Dai represented control, 0.05% quercetin group and 0.003% daidzein group, respectively. n = 6.

**Figure 3:**
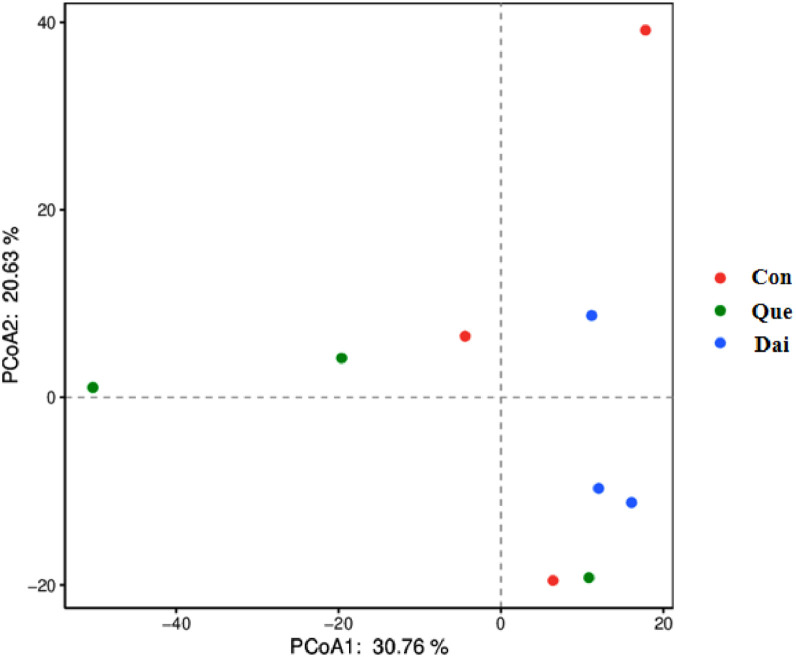
Principal co-ordinate analysis of cecal microflora in laying hens during the late laying period. Con, Que, and Dai represented control, 0.05% quercetin group and 0.003% daidzein group, respectively. n = 6. Abbreviation: PCoA, principal co-ordinate analysis.

Quercetin and daidzein did not significantly influence the alpha diversity of cecal microflora (Table 7). In addition, Shannon-Wiener rarefaction curves of each sample reflected the microflora diversity at the different sequencing quantities, the curve tended to be smooth, suggesting that sequencing data was large enough to reflect the vast majority of microflora information in cecal digesta of laying hens during the late laying period (Figure 2). OTUs Principal Co-ordinate Analysis **(PCoA)** showed that daidzein exhibited little difference among the groups. The interpretation of PCoA1 axis and PCoA2 axis were 30.76% and 20.63%, respectively (Figure 3).

**Table 7:** Effects of quercetin and daidzein on alpha diversity of cecal microflora in laying hens during the late laying period.

Items	Control	Quercetin	Daidzein	SEM	*P* value
Good's coverage	0.96	0.95	0.96	0.01	0.507
Shannon	6.85	7.20	6.58	0.25	0.118
Simpson	0.97	0.98	0.97	0.01	0.286
Chao1	850.00	885.00	795.00	98.96	0.673

Means in the same row with different letters are significantly different (*P* < 0.05). n = 6.

**Figure 4:**
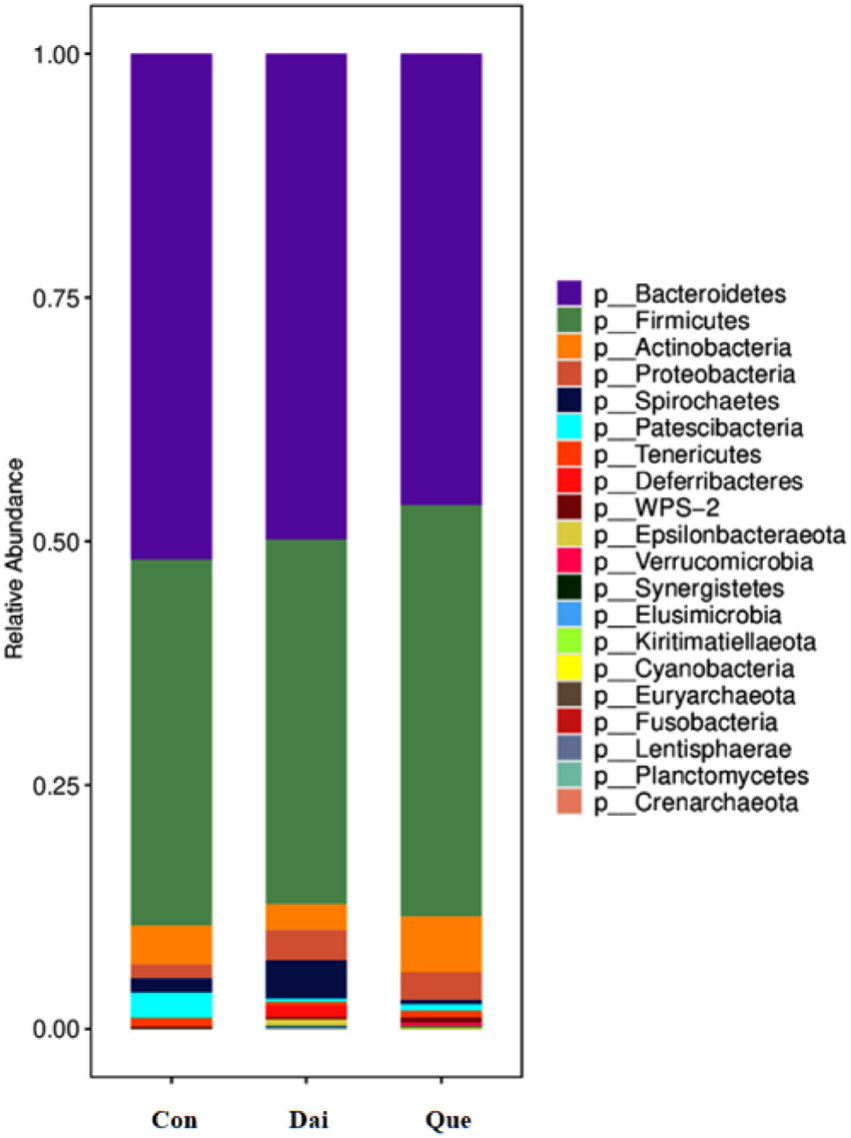
Relative abundance of cecal microflora at the phylum level in laying hens during the late laying period. Con, Que, and Dai represented control, 0.05% quercetin group and 0.003% daidzein group, respectively. n = 6.

There was no significant difference among 3 groups in the relative abundance of microflora in the cecum of laying hens during the late laying period (phylum level) (Figure 4).

**Figure 5:**
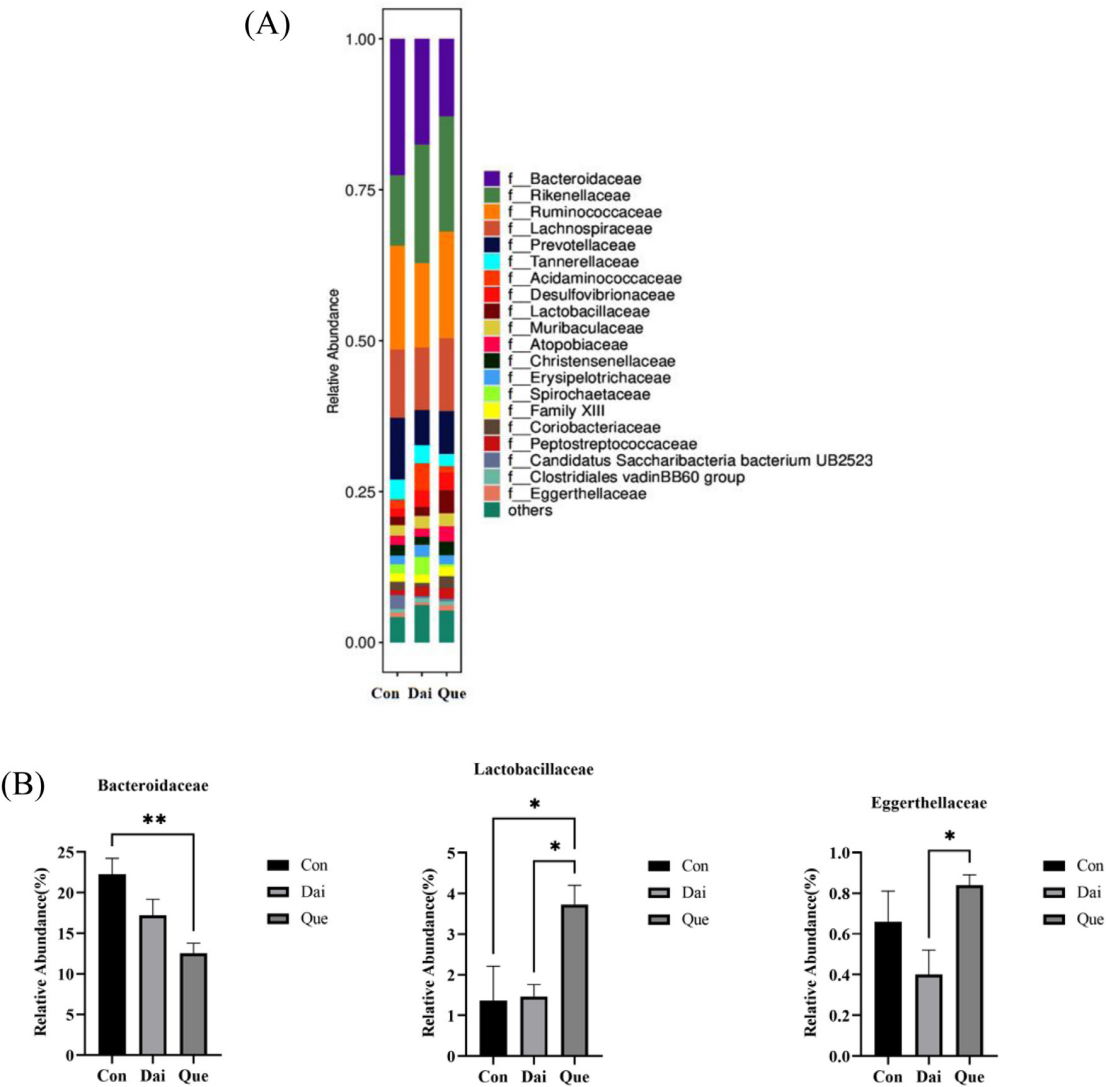
Relative abundance of cecal microflora at the family level in laying hens during the late laying period. Con, Que, and Dai represented control, 0.05% quercetin group and 0.003% daidzein group, respectively. Results are expressed as means ± SEM (n = 6). * indicated significant difference (*P* < 0.05), and ** indicated extremely significant difference (*P* < 0.01).

Compared with the control, the relative abundance of *Bacteroidaceae* was significantly decreased (*P* < 0.01), and the relative abundance of *Lactobacillaceae* was significantly increased in the quercetin group (*P* < 0.05); there was no significant difference in the daidzein group. The relative abundance of *Lactobacillaceae* and *Eggerthellaceae* in the quercetin group was significantly higher than that in the daidzein group (*P* < 0.05) (Figures 5A and 5B).

**Figure 6:**
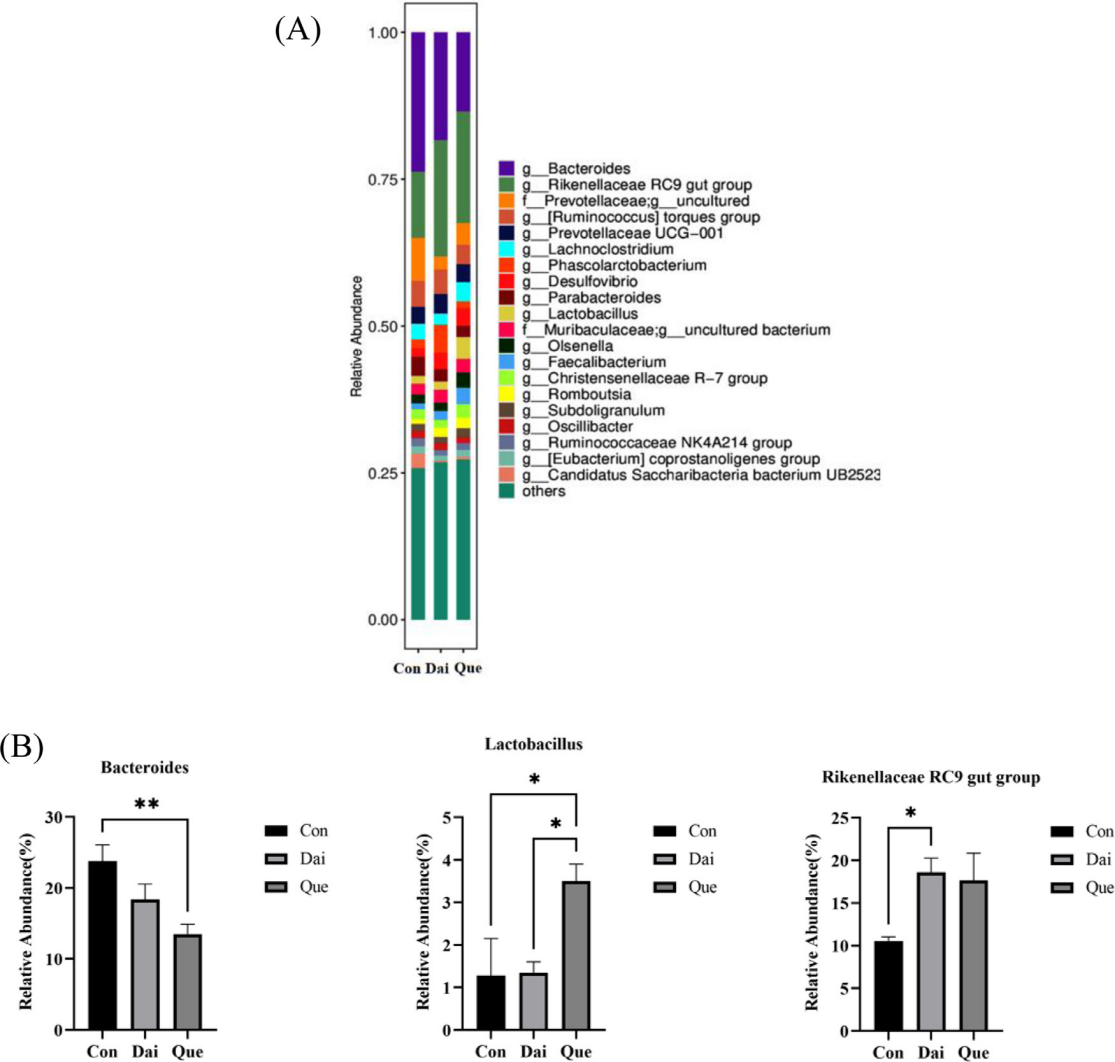
Relative abundance of cecal microflora at the genus level in laying hens during the late laying period. Con, Que, and Dai represented control, 0.05% quercetin group and 0.003% daidzein group, respectively. Results are expressed as means ± SEM (n = 6). * indicated significant difference (*P* < 0.05), and ** indicated extremely significant difference (*P* < 0.01).

Compared with the control, the relative abundance of *Bacteroides* in the quercetin group was significantly decreased (*P* < 0.01), and the relative abundance of *Lactobacillus* was significantly increased (*P* < 0.05); the relative abundance of *Rikenellaceae RC9 gut group* in the daidzein group was significantly increased (*P* < 0.05). The relative abundance of *Lactobacillus* in the quercetin group was significantly higher than that in the daidzein group (*P* < 0.05) (Figures 6A and 6B).

Compared with the control, quercetin increased the economic efficiency of production by 8.77%. Quercetin decreased total expenditure and increased economic efficiency compared with the daidzein (Table 8).

**Table 8:** Effects of quercetin and daidzein on economic efficiency of production in laying hens during the late laying period.

Items	Control	Quercetin	Daidzein
Total expenditure (CNY)	2889.04	3333.33	3485.62
Eggs income (CNY)	3431.37	3923.23	3733.90
Profits (CNY)	542.33	589.90	248.28

Basal diet: 2800.00 CNY/t, quercetin: 1.00 CNY/g, daidzein: 22.24 CNY/g. At the end of the trial, the price of eggs was 9.8 CNY/kg.

## DISCUSSION

The binding affinity of phytoestrogens daidzein and quercetin to the estrogen receptors is 200-fold and 2,500-fold lower than E_2_ (Kuiper et al., 1998), respectively. To ensure the same estrogen-like effect, the doses of 0.003% and 0.05% of daidzein and quercetin were chosen in this study by calculating the relative molecular mass of daidzein (254.24) and quercetin (338.27). Absorption and utilization of few nutrients are susceptible to the environment due to physiological characteristics in laying hens during the late laying period. Both oxidative stress and abnormal hormone secretion decrease production performance in laying hens. Therefore, in terms of production and economics, it is of significance to increase production performance in laying hens during the late laying period.

Currently, the effects of flavonoids on poultry production caught much attention. Flavonoids have a series of biological activities including anti-oxidation, regulating lipid metabolism, enhancing immune function, etc. They may also increase production performance in laying birds, thus improving the economic returns of farmers. Diets supplemented with 30 and 60 mg/kg hawthorn leaf flavonoids increased laying rate in laying hens (Dai et al., 2021). The laying rate, feed intake, and egg weight tend to increase and the ratio of feed-to-egg weight tends to decrease with dietary quercetin (400 mg/kg) supplementation in Tianfu laying hens at 52 wk old (Amevor et al., 2021). Dietary supplementation with 200 and 400 mg/kg quercetin significantly increased the laying rate and decreased the feed-to-egg weight ratio, however, did not significantly affect egg weight in laying hens (Suo, 2013). Dietary supplementation with 25 mg/kg daidzein increased the laying rate, however, did not influence egg weight in laying hens during the late laying period (Yao et al., 2007). Diet supplemented with 5 mg/kg daidzein increased the laying rate by 7.7% in ducks (Zhao et al., 2004). In the present study, the quercetin significantly increased the laying rate and decreased the feed-to-egg weight ratio, this result further confirmed that quercetin was positively correlated with production performance in laying hens. However, the daidzein did not significantly influence on production performance, the difference possibly resulted from flavonoid structure, species and physiological conditions of laying hens (Zhao et al., 2005). Additionally, our results indicated that the effect of quercetin increasing production performance was better than daidzein in laying hens during the late laying period, however, its mechanism needs to be further researched.

Production performance gradually declined with age due to ovarian senescence and hormone changes resulting from oxidative stress in laying hens (Liu et al., 2018b). Reactive oxygen species are simultaneously accumulated and scavenged by the enzymatic and nonenzymatic antioxidant system during aging (Shen et al., 2017). The antioxidant enzymes include SOD, CAT, and GSH-Px. SOD catalyzes the conversion of superoxide radicals to oxygen and hydrogen peroxide (Abreu and Cabelli, 2010). CAT and GSH-Px may convert hydrogen peroxide to water (Devine et al., 2012), meanwhile, GSH-Px also decomposes nitric oxide and peroxynitrite (Tong et al., 2015). T-AOC is a comprehensive index for evaluating the antioxidant function of the body (Cadenas and Davies, 2000). MDA is a by-product of lipid peroxidation and a major sign of oxidative stress. However, the occurrence of oxidative stress is usually accompanied by changing the activity of antioxidant enzymes in the body (Biazus et al., 2017; da Rosa et al., 2019). The activities of GSH, T-SOD, GSH-Px, and T-AOC content in liver of 580-day-old laying hens was lower than those of younger laying hens, and the MDA content was opposite (Liu et al., 2018a). It suggested that the organism was irreparably damaged by accumulating biological by-products with aging (Yin and Chen, 2005). Therefore, it is especially important to strengthen the defense and repair capabilities of laying hens.

Many studies have shown that flavonoids have anti-oxidation for alleviating oxidative stress. Diet supplemented with 3.5 to 4.5 g/kg *Ginkgo biloba* leaves (the main active ingredients of *Ginkgo biloba* leaves are flavonoids) and 250 to 1,000 mg/kg quercetin improved the growth performance and meat stability by increasing the T-SOD and T-AOC and decreasing the MDA content in the muscle of broiler chickens (Niu et al., 2017; Zhang and Kim, 2020). Hesperidin, naringin, and quercetin increased activities of SOD and GSH-Px and decreased MDA content in serum of Lohmann white laying hens at 28 wk old, and effect of quercetin was the best (Iskender et al., 2016). Quercetin decreased MDA content of liver and alleviated oxidative stress in quails (Arslan et al., 2022). Our previous study showed that 0.04% quercetin increased the antioxidant capacity by increasing T-AOC content and activities of SOD, GSH-Px in serum and SOD activity in liver, thus improved production performance in laying hens during the late laying period (Sun, 2015). Dietary supplementation with 5 and 10 mg/kg daidzein also increased laying rate by increasing T-AOC content and GSH-Px activity in liver and reducing MDA content in serum of broiler breeder hens (Ni et al., 2012). Diet supplemented with 170 mg/kg daidzein improved reproductive performance by increasing activities of GSH-Px and CAT in serum of female rabbits (Xie et al., 2022b). In addition, quercetin and daidzein protected chicken primordial germ cells (PGCs) from oxidative damage resulted from reactive oxygen species, and the effect of quercetin was better than daidzein (Tang et al., 2006).

In the present study, the quercetin increased activities of SOD and GSH-Px and reduced MDA content; daidzein increased SOD activity, reduce MDA content in serum and liver, and both quercetin and daidzein increased T-AOC content in liver of laying hens, it is similar to the other results of flavonoids (Iskender et al., 2016). It indicated that flavonoids improved antioxidant capacity, thereby delaying aging and relieving oxidation stress in laying hens. Additionally, our results indicated that the effects of quercetin increasing GSH-Px activity was better than daidzein in serum and liver. However, the up-regulation of glutathione and its related enzyme systems played an important role in antioxidant system (Choi, 2006), daidzein did not improve egg production, it possibly resulted from not increasing GSH-Px activity in serum and liver.

The growth, development, and reproduction of birds depend on the secretion and metabolism of hormones including reproductive hormones and growth hormones. It is important for the normal ovary to maintain fertility and health in females. The growth and development of ovarian follicles require a series of biochemical and physiological changes such as gonadotrophin secretion, steroid hormone synthesis, cell proliferation and differentiation in birds (Liu and Zhang, 2008). Estrogen regulates reproduction in female birds. The content of serum E_2_ may reflect estrogen status and E_2_ may stimulate follicles growth in laying hens (Zhang et al., 2021a). FSH promotes follicles growth, development, and maturation, and LH induces follicles ovulation (Guo et al., 2020a). FSH and LH synergistically regulate E_2_ synthesis in follicles. GH secreted by the pituitary does not directly promote growth in birds, however, it may indirectly work through systemic or local production of IGF-1, which promotes cell growth (Silva et al., 2009; Martins et al., 2014). Thyroid hormones and the GH-IGF-1 system may regulate growth and development of birds (Zhao et al., 2013). T_3_ plays an important role in oviduct development and is associated with growth, differentiation and development in birds (Dellovade et al., 1996; Vermaut et al., 1998; Cheserek et al., 2016). Therefore, the increase in serum hormones may improve the reproductive ability of animals.

Flavonoids with weak estrogen activity influence reproduction by regulating hormone secretion. Our previous study showed that 0.04% quercetin improved production performance by increasing content of E_2_, LH, FSH, GH and IGF-1 in serum of laying hens (Yang et al., 2018). Diet supplemented with 30 mg/kg daidzein also increased laying rate and LH content in laying hens (Shao et al., 2020). Diet supplemented with 5 mg/kg daidzein enhanced content of T_4_ and E_2_ in serum of Shaoxing ducks during the postpeak stage (Zhao et al., 2005). Diet supplemented with 10 to 30 mg/kg daidzein increased content of serum GH in Zhedong white geese during the convalescent period, which is in favor of initiating the next laying cycle as soon as possible (Zhao et al., 2013). Additionally, daidzein increased content of IGF-1 and E_2_ in serum and amniotic fluid of sows, consequently, improved embryo survival and reproductive performance in sows (Xie et al., 2020; Li et al., 2021; Xie et al., 2022a).

In the present study, quercetin increased content of E_2_, LH, FSH, GH, IGF-1, T_3,_ and T_4_; daidzein increased content of FSH, IGF-1 and T_3_ in serum of laying hens, and content of serum E_2_, LH, FSH, GH, IGF-1, T_3,_ and T_4_ in the quercetin group was higher than those of the daidzein group. It further indicated that flavonoids with estrogen-like activities, regulated the secretion of reproductive hormones (LH, FSH, and E_2_), as well as hormones of growth axis (GH, IGF-1, T_3_, T_4_), thus improved reproductive performance in laying hens. Furthermore, content of serum E_2_ reflects follicle development and egg production in birds to some extent (Leszczynski et al., 1985), daidzein did not improve egg production because of not increasing content of serum E_2_.

The ultimate aim is to improve production and maintain health and welfare of birds for the poultry industry. The gut contains a complex community of microorganisms (gut microflora), which play an important role in digestion, absorption and metabolism in laying hens (Rothe and Blaut, 2013). The composition of the gut microflora may be affected by breed, age, environment, and diet in laying hens, diet is the most important factor influencing gut microflora (Scott et al., 2013; Kers et al., 2018). Some studies proved that the low production performance is possibly related to the exhaustion of intestinal function in laying hens during the late laying period (Xu et al., 2022). The cecal microflora is complicated and flexible, including *Firmicutes, Bacteroidetes, Actinobacteria, Proteobacteria*, etc. in laying hens (Rychlik, 2020). The cecum is also the major site of microflora fermentation, the bacterial communities exceed 10^12^ per gram of digesta (Brisbin et al., 2008), which affects the health and production performance of laying hens (Zhang et al., 2021b).

The effects of quercetin and daidzein on the cecal microflora were analyzed using Illumina NovaSeq platform sequencing technology in laying hens during the late laying period. The rarefaction curve reflects the microflora diversity in cecum, the smooth curve suggested that sequencing data is large enough to reflect the vast microflora information. The Chao 1 index means the abundant species of bacteria, the lower the abundant species in the bacteria community, the higher Chao 1 index (Zhao et al., 2022). Shannon and Simpson index characterizes the diversity of bacteria community, the higher the bacteria diversity, the higher the Shannon index and the lower Simpson index (Grice et al., 2009). Our results showed that quercetin and daidzein did not affect the alpha diversity of cecal microflora in laying hens during the late laying period. The results indicated that the cecal microflora was in dynamic balance.

Flavonoids significantly affect intestinal flora. Kudzu-leaf flavonoids increased the communities of beneficial flora in Yellow-feathered broilers (Xue et al., 2021). Diet supplemented with 200 mg/kg quercetin promoted growth of broilers by ameliorating intestinal flora (Sun et al., 2022). Quercetin decreased the *Coliforms* and increased the *Bifidobacteria* and *Lactobacillus* communities in cecum, thus regulated the intestinal environment and enhanced production performance in laying hens (Liu et al., 2014; Dong et al., 2020). In addition, quercetin inhibited growth of *Staphylococcus aureus* and *Escherichia coli*, promoted the growth of *Lactobacillus* and *Bifidobacteria* to maintain intestinal health in cecum of AA broilers (Wang et al., 2018), and increased the communities of *Bacteroides, Bifidobacterium, Lactobacillus*, and *Clostridia* and decreased the communities of *Fusobacterium* and *Enterococcus*, thereby modifying intestinal flora in mice (Lin et al., 2019).

Our study showed that *Bacteroidetes, Firmicutes, Actinobacteria*, and *Proteobacteria* dominated in cecum of laying hens at the phylum level, however, were not affected by quercetin and daidzein (Fu et al., 2018). The quercetin significantly increased the relative abundance of *Lactobacillaceae* and *Lactobacillus* and decreased the relative abundance of *Bacteroidaceae* and *Bacteroides* at the family and genus levels; daidzein increased the relative abundance of *Rikenellaceae RC9 gut group* at the genus level. The quercetin increased the relative abundance of *Lactobacillaceae* and *Lactobacillus* at the family and genus levels, compared with the daidzein. The previous studies reported that *Rikenellaceae RC9 gut group* and *Bacteroides* were correlated with the body health (Zhou et al., 2018; Guo et al., 2022), *Lactobacillaceae* and *Lactobacillus* may modify growth and reproduction of broilers. Choe et al. 2012 found that 0.6% of *Lactobacillus plantarum* increased laying rate in laying hens (Choe et al., 2012), this is consistent with our results that the laying rate and the relative abundance of *Lactobacillus* in quercetin treatment was higher than those in control and the daidzein. Probiotic effects of *Lactobacillus* enhanced intestinal immunity and inhibited growth of pathogens (Balcazar et al., 2006). These results indicated that quercetin enhanced production performance by increasing the relative abundance of *Lactobacillaceae* and *Lactobacillus* at the family and genus levels in cecum of laying hens, and quercetin was more effective than daidzein.

The economic efficiency only included feeding costs under the same other conditions in this experiment. Our results showed that the quercetin increased economic efficiency compared with the control, meanwhile, diet supplemented with quercetin increased egg quality and decreased total cholesterol content in eggs (Simitzis et al., 2018), which promoted cardiovascular health, won broad consumer acceptance, sold higher prices, thereby higher economic efficiency will be achieved. The quercetin decreased feeding costs and increased economic efficiency compared with the daidzein. It indicated that quercetin substituted for daidzein will increase the economic efficiency in laying hens during the late laying period.

## CONCLUSION

In conclusion, quercetin increased production performance and economic efficiency through improving antioxidant state, increasing secretion of hormones related to reproduction and growth, and regulating cecal microflora in laying hens during the late laying period; quercetin was more effective than daidzein under this experimental condition.

## ACKNOWLEDGMENTS

The authors thank the National Natural Science Foundation of China (32072749), Chenguang Biotech Group Co. Ltd and Hebei Innovative Technology Center of Plant-derived Animal Nutrients (YF-07-HE171) for funding this work.

Ethics Statement: The animal study was reviewed and approved by Laboratory Animal Control Committee of Northeast Agricultural University (NEAUEC20200203).

## DISCLOSURES

The authors declare no conflicts of interest.
